# Low glycosylated ferritin is a sensitive biomarker of severe COVID-19

**DOI:** 10.1038/s41423-020-00544-0

**Published:** 2020-09-11

**Authors:** Maxime Fauter, Sébastien Viel, Sabine Zaepfel, Pierre Pradat, Julie Fiscus, Marine Villard, Lorna Garnier, Thierry Walzer, Pascal Sève, Thomas Henry, Yvan Jamilloux

**Affiliations:** 1 CIRI (International Centre for Research in Infectiology), Inserm U1111, Université Claude Bernard Lyon 1, CNRS, UMR5308, ENS de Lyon, Lyon, France; 2 Department of Internal Medicine, University Hospital Croix-Rousse, Lyon 1 University, Lyon, France; 3 Immunology Laboratory, University Hospital Lyon Sud, Lyon 1 University, Lyon, France; 4 LIFE, Lyon Immunology Federation, Lyon, France; 5 Biochemistry Laboratory, University Hospital Croix-Rousse, Lyon 1 University, Lyon, France; 6 Centre for Clinical Research, University Hospital Croix-Rousse, Hospices Civils de Lyon, Lyon, France

**Keywords:** Diagnostic markers, Prognostic markers

Severe forms of coronavirus disease 2019 (COVID-19) have been associated with a cytokine storm mainly involving interleukin (IL)-6, IL-1β, and TNF.^1,2^ Several authors have reported features of macrophage activation, thus comparing the cytokine storm of COVID-19 to reactive hemophagocytic lymphohistiocytosis (reHLH).^3,4^ However, these data have been balanced by other studies primarily involving IL-6 and, therefore, a mechanism closer to the complex immune dysregulation observed in sepsis.^5,6^ Considering these discrepancies, serum cytokine profiling may not be the best option for assessing COVID-19 severity and prognosis.

Serum ferritin, an inflammatory biomarker, is elevated in most COVID-19 patients and has been correlated with severity and mortality.^7^ The measurement of the glycosylated fraction of ferritin (GF), which could be readily implemented in routine diagnosis, is of great interest in the diagnosis of reHLH (and in Still’s disease, which is frequently associated with macrophage activation syndrome).^8,9^ Indeed, a GF rate < 25% has a positive predictive value of 88% and a negative predictive value of 100% for reHLH.^8^

Here, we assessed whether the GF rate could serve as a biomarker for COVID-19 severity and prognosis.

We prospectively analyzed the immunobiochemical parameters of 58 patients with COVID-19 who were hospitalized in the Internal Medicine Department from March 21 to May 9, 2020. All patients had confirmed SARS-CoV-2 infection. Patient characteristics are shown in Table S1. The male/female sex ratio was 1.9, and the mean age was 73.3 (range, 39–99) years. The median time from symptom onset to hospital admission (i.e., first biomarker evaluation) was 8.6 (range, 3–19) days. Upon admission, acute phase reactants were elevated [either C-reactive protein ≥ 5 mg/L, procalcitonin (PCT) ≥ 0.5 μg/L, and/or fibrinogen ≥ 4.2 mg/L] in all but one patient. Lymphopenia was reported in 46% of the patients (mean, 1056 ± 502/mm^3^).

During follow-up, 17 patients had severe worsening of their condition (see “Supplemental Methods”), and seven patients died. Thus, the fatality rate was 12.1%.

Patients who required oxygen with a flow rate > 3 L/min had significantly higher ferritin levels (4843 vs. 640 μg/L, *p* < 0.001) along with a diminished GF rate (30.9 vs. 42.8%, *p* = 0.0018). PCT, AST, and ALT were also significantly increased, while the lymphocyte levels were significantly lower (Table S2 and Fig. S1).

Patients with severe COVID-19 had significantly lower levels of lymphocytes and hemoglobin. Leukopenia and thrombopenia, as well as increased levels of PCT, AST, ALT, and ferritin, were significantly more frequent. In these patients, the GF rate was significantly lower (31.6 vs. 41.6%, *p* = 0.0162).

Patients who succumbed to COVID-19 had significantly less serum hemoglobin, platelets, and lymphocytes. Leukopenia and PCT elevation were also significantly associated with progression to death. The GF rate was not significantly different between dead and live patients (30.1 vs. 38.8%, *p* = 0.143).

In all these analyses, neither the lymphocyte-to-neutrophil ratio nor the platelet-to-neutrophil ratio was different between groups (data not shown).

We then assessed the ability of certain parameters to serve as biomarkers of disease severity and outcome (Table 1) by constructing ROC curves and calculating the area under the curve (AUC). Among the biomarkers, PCT had the highest AUC for disease severity (AUC = 0.787) and oxygen requirement (AUC = 0.836), whereas lymphocyte count had the highest AUC for death prediction (AUC = 0.861). However, with a cut-off value set at 40%, GF rate had the best sensitivity of all these outcomes (>94%), although it displayed a low specificity (range, 43–55%). Alternatively, the lymphocyte decrease (under 540/mm^3^) was highly specific (>92%) to severe forms but less sensitive (range, 42–71%). PCT assessment yielded good specificity (>82%) but poor sensitivity (range, 46–56%). Interestingly, for most of these outcomes, assessment of the GF rate was superior to assessment of serum ferritin alone.

**Table 1:** Biomarker values

Biomarker	AUC (95% CI)	Cut-off value	Sensitivity (%)	Specificity (%)	PPV (%)	NPV (%)
O2 requirement >3 L/min						
PCT (μg/L)	**0.836 (0.724, 0.948)**	0.5	56.3	94.3	81.8	82.5
Ferritin (μg/L)	0.791 (0.659, 0.922)	772	75.0	68.4	55.6	83.9
Glycosylated ferritin rate (%)	0.745 (0.619, 0.872)	40	**95**	55.3	52.8	**95.5**
AST (IU/L)	0.754 (0.616, 0.892)	38	70.0	71.1	56.0	81.8
ALT (IU/L)	0.759 (0.635, 0.882)	40	65.0	76.3	59.1	80.6
Lymphocyte count (/mm^3^)	0.718 (0.572, 0.863)	540	42.1	**97.4**	**88.9**	77.1
Hemoglobin (g/dL)	0.645 (0.482, 0.808)	12.1	60.0	63.2	46.2	75
Disease severity						
PCT (μg/L)	**0.787 (0.655, 0.919)**	0.5	46.2	86.8	54.6	82.5
Ferritin (μg/L)	0.709 (0.554, 0.864)	772	64.7	60.9	40.7	80.7
Glycosylated ferritin rate (%)	0.698 (0.563, 0.837)	40	**94.1**	51.2	44.4	**95.5**
AST (IU/L)	0.670 (0.510, 0.830)	38	58.8	63.4	40	78.8
ALT (IU/L)	0.725 (0.591, 0.860)	40	64.7	73.2	50	83.3
Lymphocyte count (/mm^3^)	0.728 (0.570, 0.885)	540	50	**97.6**	**88.9**	83.3
Hemoglobin (g/dL)	0.718 (0.553, 0.883)	12.1	70.6	68.3	48.0	84.9
Progression to death						
PCT (μg/L)	0.759 (0.576, 0.943)	0.5	50.0	82.2	27.3	92.5
Ferritin (μg/L)	0.658 (0.394, 0.923)	772	57.1	54.9	14.8	90.3
Glycosylated ferritin rate (%)	0.674 (0.486, 0.861)	40	**100**	43.1	19.4	**100**
AST (IU/L)	0.657 (0.424, 0.889)	38	57.1	58.8	16.0	90.9
ALT (IU/L)	0.679 (0.488, 0.870)	40	57.1	64.7	18.2	91.7
Lymphocyte count (/mm^3^)	**0.861 (0.728, 0.995)**	540	71.4	**92.0**	**55.6**	95.8
Hemoglobin (g/dL)	0.779 (0.529, 1.0)	12.1	85.7	62.8	24	96.9

Bold font indicates the best biomarker

*AUC* area under the curve, *PPV* positive predictive value, *NPV* negative predictive value, *BMI* body mass index, *CRP* C-reactive protein, *PCT* procalcitonin, *AST* aspartate aminotransferase, *ALT* alanine aminotransferase

Among all these biological parameters, the only correlation observed was between the GF rate and lymphocyte count (*r* = 0.564, *p* < 0.0001, Fig. S2).

We then assessed the blood levels of a panel of cytokines eventually involved in the COVID-19 immune response^1,2,7^ and tested their value as severity biomarkers. Cytokine levels exceeded the upper reference limit in the majority of the patients (62% for IFN-γ, 98% for IL-6, 97% for TNF-α, 63% for IL-18, 82% for MCP-1, 71% for IL-10, and 96% for IL-1Ra and sIL-2R). Except for IL-10, we found no significant differences in cytokines between the groups of patients (Fig. S4). By analyzing the cytokine profiles, we found that 40% were evocative of macrophage activation (i.e., elevation of at least two cytokines among IL-1Ra, IL-18, or sIL-2R), 13% were marked by IL-6 predominance, 38% were compatible with global hypercytokinemia, and 9% were not specific.

Last, we analyzed the kinetics of several parameters in a subset of patients (*n* = 18) who underwent repeated blood sampling during the disease course (Fig. S2). Although, due to the limited number of patients, we did not find significant profiles, we observed a trend toward an inverse correlation between GF rate and both IL-6 and IL-10 levels, with values reaching their nadir or maximum between days 7 and 11.

Overall, our results indicate that patients with COVID-19 have heterogeneous cytokine profiles, precluding the use of such profiles as severity or prognosis biomarkers. Conversely, routine biomarkers, such as the PCT or lymphocyte count, had a better specificity than cytokines did but displayed a low sensitivity. The GF rate is a highly sensitive biomarker and better discriminates severe patients than ferritin alone. Thus, the GF rate should be evaluated for its prognostic value in a larger cohort. A combination of the lymphocyte count (<540/mm^3^), PCT (≥0.5 μg/L), and GF rate (≤40%) could be the basis of a severity score (one point each) that guides clinical decision making. In our cohort, a score ≥ 2 would provide a sensitivity of 71% and a specificity of 95% for disease severity. However, the risk of progression to death remains better evaluated by lymphopenia alone.

## Supplementary information

Supplemental material
