# Supplementary material for: Low glycosylated ferritin is a sensitive biomarker of severe COVID-19
Source: Cell Mol Immunol. 2020 Sep 11;17(11):1183–5. doi: 10.1038/s41423-020-00544-0 (PMC7484604; doi:10.1038/s41423-020-00544-0)
Supplement: Supplementary file 1 — Supplemental material [file 41423_2020_544_MOESM1_ESM.docx]

**SUPPLEMENTARY MATERIAL**

related to:

**Low glycosylated ferritin is a sensitive biomarker of severe COVID-19**

by: Maxime Fauter, Sébastien Viel, Sabine Zaepfel, Pierre Pradat, Julie Fiscus, Marine Villard, Lorna Garnier, Thierry Walzer, Pascal Sève, Thomas Henry, Yvan Jamilloux

**EXTENDED METHODS**

***Patient recruitment and severity assessment***

We included all consecutive patients hospitalized in our Department of Internal Medicine (Hospices Civils de Lyon, France) between March 21 and May 9, 2020 for confirmed SARS-CoV2 infection. COVID-19 was confirmed either by PCR analysis of nasal swabs or by typical findings on chest computed tomography.

No asymptomatic patient was included in the study.

The disease was considered severe if at least one of the following criteria was present:

- oxygen requirement with flow rate >6L/min,

- respiratory rate >30/min or <10/min or symptoms of acute respiratory distress,

- extra-pulmonary organ failure.

Mild disease was defined as non-severe disease.

Given the exceptional health situation, not all the patients who needed to be admitted to intensive care unit were effectively transferred. Some (8/17, 47%) were therefore treated in conventional units.

***Ferritin and glycosylated ferritin determination***

Total serum ferritin concentration was measured on a SIEMENS Dimension® Vista™1500 using a homogeneous immunoassay method: LOCI (Luminescent oxygen channeling assay). Glycosylated ferritin, expressed as the percentage of total serum ferritin, was determined using LOCI method after separation on a concanavaline A-sépharose 4B gel (Amersham Biosciences®).

***Cytokine measurement***

Cytokine concentrations were measured by Ella technology (Protein Simple®), following manufacturer’s instructions.

***Ethics***

The work described has been carried out in accordance with The Code of Ethics of the World Medical Association (Declaration of Helsinki). The FERRIGLY COVID study was approved by an ethical committee for biomedical research (*Comite de Protection des Personnes HCL*) under the number 20-55. The study was declared to the French National Data Protection Agency (Commission Nationale de l'Informatique et des Libertés, CNIL) and registered under the number 20-235. All the included patients were informed and the list of opposed patients was carefully checked. None of the patients included declared their opposition to the study.

The study was registered on www.clinicaltrials.gov under the unique identifier NCT04469153.

***Statistical analysis***

Quantitative variables are expressed as means (with range or standard deviation in the text, and with SEM in the figures) and were compared between groups using the non-parametric Mann–Whitney test. Categorical variables are expressed as numbers and percentages and were compared between groups by performing the Chi-squared test or the Fisher's exact test as appropriate. P values were considered significant when they were <0.05. The ability of each biomarker to discriminate between patient groups was quantified by the construction of ROC curves and the estimation of areas under the curve (AUC) with their associated 95%-confidence intervals (95%-CI). From the ROC curves, the threshold maximizing the number of well-classified subjects was estimated by maximizing the Youden index. Sensitivity, specificity, positive and negative predictive values were calculated using this threshold (e.g. lymphocyte count, haemoglobin, ferritin, glycosylated ferritin) or using consensual thresholds (i.e. reference values, for PCT, AST, and ALT). Correlations between continuous data were tested by calculating the Pearson correlation coefficient (and its associated 95%-confidence interval). Statistical analyses were carried out with Prism (GraphPad, v6) and R software (R Foundation for Statistical Computing, v4.0, http://www.R-project.org).

**Table S1: Description of the study population**

| Parameters | Mean (±SD) or n (%) |
| --- | --- |
| Clinical  - age (years)  - sex (n, men/women)  - BMI (kg/m²)  - BMI >25 (n, %)  - BMI >30 (n, %)  - Pre-existing conditions (n, %)  - Hypertension (n, %)  - Diabetes (n, %)  - Delay symptom onset to sampling (days)  - Respiratory rate 22≤x<30/min (n, %)  - Respiratory rate ≥30/min (n, %)  - No oxygen (n, %)  - Oxygen (with flow rate ≤3L/min; n, %)  - Oxygen flow rate >3L/min (n, %)  - qSOFA (mean, min-max) | 73.3 (14.7)  38/20  26.22 (5.66)  31/58 (53%)  10/58 (17%)  45 (78%)  26 (45%)  13 (22%)  9.2 (3.7)  15 (26%)  9 (16%)  14 (24%)  24 (41%)  20 (34%)  0.45 (0.57) |
| Laboratory tests  - Leucocytes (/mm3)  - Lymphocytes (/mm3)  - Haemoglobin (g/dL)  - CRP (mg/L)  - Fibrinogen (g/L)  - PCT (μg/L)  - Ferritin (μg/L)  - Glycosylated ferritin (% of total ferritin)  - D-dimers (g/L)  - Prothrombin time  - AST  - ALT | 6433 (3092)  1056 (502)  12.3 (2.3)  80.0 (70)  5.19 (1.67)  1.32 (5.27)  2089 (7949)  39.0 (14)  1759 (1833)  78.3 (15.8)  82 (190)  59 (111) |
| Imaging (32/58)  - abnormal chest CT <50% (n, %)  - abnormal chest CT >50% (n, %) | 26/32 (81%)  6/32 (19%) |
| Outcome  - ICU requirement (n, %)  - ICU admission (n, %)  - Death (n, %) | 17 (29%)  9 (16%)  7 (12%) |

BMI: body mass index; CRP: C-reactive protein; PCT: procalcitonin; AST: aspartate-aminotransferase; ALT: alanine-aminotransferase; CT: computed tomography; ICU: intensive care unit. Bold font indicates P-value <0.05.

**Table S2:** **Comparison of patient data according to oxygen requirement, COVID-19 severity and outcome**

| Parameter |  |  | *P-value* |
| --- | --- | --- | --- |
| O2 requirement >3L/min | **O2 flow rate ≤3L/min**  **n=38** | **O2 flow rate >3L/min**  **n=20** |  |
| - mean age (years)  - mean BMI (kg/m²)  - pre-existing conditions (%)  - hypertension (%)  - diabetes (%)  - fever (T >38.4°C, %)  - respiratory rate >22/min (%)  - leukopenia (%)  - lymphocyte count (/mm3)  - haemoglobin (g/dL)  - platelets (/mm3)  - CRP (mg/L)  - fibrinogen (g/L)  - PCT (μg/L)  - ferritin (μg/L)  - glycosylated ferritin rate (% of total ferritin)  - d-dimers (g/L)  - AST (IU/L)  - ALT (IU/L) | 75.0  26.2  84.2  57.9  26.3  13.2  18.4  10.5  1185  12.7  271,180  68.9  5.1  0.20  640  42.8  1736  39  36 | 69.9  25.7  65.0  20.0  15  40.0  85  25.0  799  11.6  224,650  101.8  5.4  3.76  4843  30.9  1800  166  103 | 0.215  0.744  0.111  **0.011**  0.509  **0.043**  **<0.001**  0.251  **0.007**  0.072  0.369  0.205  0.231  **<0.001**  **<0.001**  **0.002**  0.082  **0.012**  **0.010** |
| Disease Severity | **Mild**  **n=41** | **Severe**  **n=17** |  |
| - mean age (years)  - mean BMI (kg/m²)  - pre-existing conditions (%)  - hypertension (%)  - diabetes (%)  - fever (T >38.4°C, %)  - respiratory rate >22/min (%)  - oxygen flow rate >3L/min (n, %)  - leukopenia (%)  - lymphocyte count (/mm3)  - haemoglobin (g/dL)  - platelets (/mm3)  - CRP (mg/L)  - fibrinogen (g/L)  - PCT (μg/L)  - ferritin (μg/L)  - glycosylated ferritin rate (% of total ferritin)  - d-dimers (g/L)  - AST (IU/L)  - ALT (IU/L) | 73.4  26.6  78.0  53.7  21.9  17.0  24.4  9.8  7.3  1168  12.8  271,590  77.3  5.2  0.25  727  41.6  1680  43  37 | 73.1  24.7  76.5  23.5  23.5  41.1  82.4  94.1  35.3  770  11.2  215,470  86.3  5.3  4.43  5374  31.6  1948  177  112 | 0.943  0.271  1  **0.046**  1  0.089  **<0.001**  **<0.001**  **0.014**  **0.007**  **0.009**  0.192  0.837  0.999  **0.001**  **0.012**  **0.016**  0.616  **0.043**  **0.007** |
| Disease outcome | **Alive**  **n=51** | **Dead**  **n=7** |  |
| - mean age (years)  - mean BMI (kg/m²)  - pre-existing conditions (%)  - hypertension (%)  - diabetes (%)  - fever (T >38.4°C, %)  - respiratory rate >22/min (%)  - oxygen flow rate >3L/min (n, %)  - leukopenia (%)  - lymphocyte count (/mm3)  - haemoglobin (g/dL)  - platelets (/mm3)  - CRP (mg/L)  - fibrinogen (g/L)  - PCT (μg/L)  - ferritin (μg/L)  - glycosylated ferritin rate (% of total ferritin)  - d-dimers (g/L)  - AST (IU/L)  - ALT (IU/L) | 71.9  26.6  76.5  47.1  19.6  23.5  37.3  27.5  9.8  1127  12.6  275,020  77.7  5.2  1.04  1955  38.8  1792  66  47 | 83  21.9  85.7  28.5  42.9  14.3  71.4  85.7  57.1  553  10.3  110,290  95.7  4.9  3.37  3062  30.1  1454  205  149 | 0.076  **0.016**  1  0.442  0.179  1  0.114  **0.005**  **0.008**  **0.001**  **0.015**  **<0.001**  0.856  0.246  **0.036**  0.186  0.143  0.631  0.187  0.129 |

BMI: body mass index; CRP: C-reactive protein; PCT: procalcitonin; AST: aspartate-aminotransferase; ALT: alanine-aminotransferase. Bold font indicates P-value <0.05.

**Fig S1:** **Comparison of biochemical parameters according to disease severity and outcome.** Each parameter was compared between patient groups according to: 1) oxygen (O2) flow rate with a cut-off set at 3L/min (left column); 2) clinical severity (central column); and 3) disease progression (right column). The p-value is indicated when it was <0.05 (Mann-Whitney test); ns means that the difference was not significant. PCT: procalcitonin; CRP: C-reactive protein; AST: aspartate-aminotransferase; ALT: alanine-aminotransferase.

**Fig S2: Lymphocyte decrease is correlated with decreased glycosylated ferritin rate.** Correlations were tested for all the immunobiochemical parameters cited in the main text, by assessing the Pearson correlation coefficient. The only significant correlation observed was between lymphocyte count and glycosylated ferritin (r=0.5641, 95% CI: 0.356-0.719, p<0.0001).

**Fig S3: Comparison of cytokine levels according to disease severity and outcome.** Blood cytokine levels were compared between patient groups according to: 1) oxygen (O2) flow rate with a cut-off set at 3L/min (left column); 2) clinical severity (central column); and 3) disease progression (right column). The p-value is indicated when it was <0.05 (Mann-Whitney test); “ns” means that the difference was not significant. IL: interleukin; IL-1Ra: IL-1 receptor antagonist, TNF: tumor necrosis factor; IFN: interferon, MCP-1: monocyte chemoattractant (=CCL2), sIL-2R: soluble IL2 receptor (= sCD25).

**Fig S4: Kinetics of immuno-biochemical parameters over COVID-19 course.** Eighteen patients had repeated (≥2) blood sampling and dosages of immuno-biochemical parameters. Data are depicted as mean (±SEM) according to time period after symptom onset (day 1-6 / day 7-11 / day 12-19). Glycosylated ferritin rates and interleukin (IL)-6 levels are also plotted as individual data (upper right panels); the connected dots represent a single patient.
